# COVID-19 and neurological training in Europe: from early challenges to future perspectives

**DOI:** 10.1007/s10072-020-04723-9

**Published:** 2020-09-24

**Authors:** Matthijs van der Meulen, Nina N. Kleineberg, David R. Schreier, David García-Azorin, Francesco Di Lorenzo

**Affiliations:** 1 Department of Neuro-Oncology, Erasmus MC Cancer Institute, Brain Tumor Center, University Medical Center Rotterdam, Dr. Molewaterplein 40, 3015 Rotterdam, GD Netherlands; 2 Department of Neurology, Faculty of Medicine, University Hospital of Cologne, University of Cologne, Cologne, Germany; 3 Cognitive Neuroscience, Institute of Neuroscience and Medicine (INM-3), Research Centre Jülich, Jülich, Germany; 4 Department of Neurology, Inselspital, Bern University Hospital, University of Bern, Bern, Switzerland; 5 Headache Unit, Department of Neurology, Hospital Clínico Universitario, Valladolid, Spain; 6 Noninvasive Brain Stimulation Unit, Scientific Institute for Research, Hospitalisation and Health Care Santa Lucia Foundation, Rome, Italy

**Keywords:** COVID-19, Residency training, Telemedicine

## Abstract

The worldwide SARS-CoV-2 pandemic is dramatically affecting health systems with consequences also for neurological residency training. Here we report early experiences and challenges that European neurologists and residents faced. The breadth of the pandemic and the social restrictions induced substantial modifications in both inpatient and outpatient clinical care and academic activities as well, adversely affecting our residency training. On the other hand we see also opportunities, such as gaining more clinical and professional skills. All these drastic and sudden changes lead us to reconsider some educational aspects of our training program that need to be improved in order to better prepare the neurologists of the future to manage unexpected and large emergency situations like the one we are living in these days. A reconsideration of the neurological training program could be beneficial to guarantee high standard level of the residency training in this period and beyond.

## Introduction

The severe acute respiratory syndrome coronavirus 2 (SARS-CoV-2) is causing a pandemic of coronavirus disease 2019 (COVID-19) and is dramatically challenging the medical workforce and healthcare systems. The sudden need for capacities to accept the huge number of infected patients and the greater exposure of healthcare workers to the virus [1] has altered the normal organization of hospitals [2].

Although neurologists are not the designated professionals working in the frontline of this emergency, the devastating breadth of the pandemic is causing profound modifications in the routine practice of neurological patient care and as a result of the neurological residency training. The situation differs between countries and even between hospitals within one country; the rapid large-scale spread and the severe restrictions regarding social life changes are diffusely affecting the neurological residency training.

Since future COVID seasonal outbreaks are forecasted [3], we aim to highlight the challenges faced by residents during the “early period” and their consequences on the training, in order to envision future opportunities to guarantee high standard level of residency training in this period and beyond.

## Challenges on neurology residency training

National and international courses and conferences are canceled or have been replaced with virtual conferences or digital platforms (for small group lessons). Although opportunities for online education exist, technical and interactive boundaries are still too prominent to replace physical presence.

Bedside teaching is deeply reduced: neurology departments have canceled almost all elective hospitalization, a golden source for residents to fully study and manage many chronic neurological disorders. Additionally, outpatient visits are strongly reduced. Apart from case emergencies, most outpatient appointments for new referrals were postponed. Therefore, physical examination cannot be performed while it is fundamental for patients’ diagnosis and management. Follow-up visits for chronic conditions were performed via telemedicine [3], challenging with elderly and those with cognitive impairments. “Learner positions” normally embarked as inner training rotations within the neurology department were stopped to reduce personnel contacts as a potential spread of the coronavirus.

Another consequence is the strong reduction of performed neurophysiological examinations. Clinical neurophysiology is a fundamental part of the neurological curriculum and its rotation to focus on performance and interpretation of all investigations. The reduction of the abovementioned activities resulted in a paradoxical lower demand for medical staff. Consequently, some residents were sent home to be “stand by.” Conversely, in the more affected areas, part of the workers stay at home preventively in case some get infected. In the most affected areas, the increasing number of COVID-19 patients and the lack of medical staff (infected or quarantined) induced profound changes to the workforce management [2]. In North Italy and Spain, bed capacity has been reserved for medical emergencies, and neurologists (including residents) have been reallocated to internal medicine or intensive care units (ICU) wards forming multidisciplinary teams [4]. Such measures have also been applied in less affected regions as a precaution, to prepare for a possible scenario in which more workforce is needed. For residents’ support, many hospitals are offering freely accessible webinars and online courses regarding the use of a ventilator machine, general knowledge on internal medicine, and pulmonology. In Spain a handbook on Neuro-COVID-19 has been rapidly produced by the National Neurological Society [5]. However, learning via online courses cannot replace structured bedside teaching, which might lead to overstrained residents, not ready to embark on their new duty.

Furthermore, we are experiencing also that the physical isolation of admitted patients, not allowed to welcome visitors, is strongly affecting their moral. This demands additional mental support and care to the patients and to their relatives, which has to be provided by residents who are usually not sufficiently trained for.

Finally, the current situation is also compromising research and clinical trials. Most of the research projects have been interrupted, and the few ongoing trials need peculiar care.

## Opportunities and future perspectives

Even though the current situation is far from ideal for patient care and neurology training, it also offers opportunities.

Neurology residents who are currently working on the ICU or COVID wards will gain intensive experience in emergency care and internal medicine, strengthening the coherence within the healthcare system beyond disciplines and professions. This crisis also yields the development of technological and strategic skills, such as advancing in telemedicine, new teaching, and meeting platforms. These skills as health advocates and collaborators are also advised by the Canadian model for medical training [6]. We cannot oversee the entire impact of this pandemic yet, but we must conclude that this sudden disease outbreak has hit the health systems hard, and lessons should be learned for future challenges. Since many (aspiring) neurologists are needed in such an extraordinary situation, neurology training programs might benefit from some structural modifications.

In most, but not all European countries, a rotation to internal medicine is mandatory [7]; neurology residents are usually not sufficiently trained to manage critically ill patients on ICUs. So, more allocated time for internal and emergency medicine in neurology training programs might be beneficial. Psychological support and communication skills are important aspects of our profession and should therefore also be incorporated in our training.

Telemedicine and digital technology represent a valid alternative in the future to consult patients who are struggling to reach the hospital [8]. Indeed, healthcare systems are planning to use digital technology more broadly: in 2020 we have a wide range of technologic support (virtual clinics, e-learning platforms, telemedicine consultations, artificial intelligence-based triage systems) [9] that can be used to enhance public health strategies and educational activities. This aspect fits well with chronic neurological diseases, and a specific training on the use, advantages, and disadvantages of digital technology should be encouraged.

For this year residents’ cohort, we recommend that missed rotations should be recovered.

## Conclusion

The COVID-19 pandemic severely and diffusely affects neurological residency training. Despite the situation and the neurological training programs in Europe differ widely, the problems faced by residents in this pandemic are similar. It also leads to new opportunities, to broaden our overall medical skills and to develop new strategies on how to remotely treat patients. In order to be prepared for future outbreaks, there is an urge to reconsider some aspects of training programs. We think that considering all these aspects would sensitize the national and international institutions to improve and adhere to standards of neurological training across borders.
